# Supplementary material for: Evaluation of the Safety, Tolerability and Immunogenicity of ShigETEC, an Oral Live Attenuated Shigella-ETEC Vaccine in Placebo-Controlled Randomized Phase 1 Trial
Source: Vaccines (Basel). 2022 Feb 21;10(2):340. doi: 10.3390/vaccines10020340 (PMC8879453; doi:10.3390/vaccines10020340)
Supplement: Supplementary file 1 [file vaccines-10-00340-s001.zip › vaccines-1562178-supplementary.pdf]

Table S1. Girardi et al.: Inclusion and Exclusion criteria for Phase 1 study with oral ShigETEC

**Inclusion criteria**	
1.	Generally healthy male and non-pregnant, non-nursing female adults aged 18 to 45 years
2.	Who were determined by medical history, physical examination and clinical judgment to be eligible for this study;
3.	Who provided written informed consent after the nature of the study had been explained;
4.	Who were available for the two to three months duration of follow-up (from enrolment to study completion);
5.	Who were available to be interviewed by study staff for the 2-month post-immunization follow-up.
**Exclusion Criteria**	
1.	Subjects who were perceived to be unavailable or difficult to contact for evaluation or study visits during the study period;
2.	Who had a known or suspected disease of cancer (excluding healed skin lesions), the immune system, or those who were receiving immunosuppressive therapy, including systemic corticosteroids or cytotoxic agents;
3.	Who had received any blood products, including immunoglobulin, in the period from six months prior to vaccination or were anticipated to receive such products through to the conclusion of the study;
4.	Who had ever received a cholera vaccine;
5.	Who were receiving antibiotics or completed antibiotic therapy in previous 7 days;
6.	Who had or were participating in or have concluded participation in a clinical research study in the last 30 days or had not cleared the experimental product or the biological effects of such experimental products prior to vaccination or received a licensed vaccine in the 30 days prior to vaccination;
7.	Who had a chronic non-gastrointestinal medical condition (e.g. hypertension, hyperlipidemia) that is not well controlled with medication;
8.	Who had a significant history of or current diagnosis of diseases of the gastrointestinal tract (i.e. liver, gallbladder, bowel, or stomach), were on medication for such disease, or had had significant bowel surgery (with the exclusion of hernia repair or appendectomy);
9.	Who had significant abnormality of blood chemistry, hematology, or screening tests (including tests for hepatitis B, HIV, HCV);
10.	Who had a history of reactive arthritis following GI infection;
11.	Who expected to work in the subsequent 2 weeks as a food handler or in direct patient, child day care, or elder care;
12.	Who had immunocompromised household member;
13.	Who had ever had a diagnosed Shigella or ETEC infection;
14.	Who had developed symptoms of Shigella or ETEC infections after having travelled to Shigella or ETEC endemic areas in the past 12 months;
15.	Who had any condition which in the opinion of the investigator put the subject at risk of non-compliance with the protocol;
16.	Who had known allergy to quinolone or azithromycin that would be used in the study;
17.	Who were HLA-B27 positive (a possible risk factor for reactive arthritis following naturally-acquired bacterial gastrointestinal infection);
18.	Who were dependent of the Sponsor, of the investigational team or his/her immediate family, or were medical students at the Clinical Trial Study Site in Debrecen;
19.	Who were living in community (i.e. students living in a dormitory);
20.	Who were positive for CoVID19 infection by PCR testing prior to randomization.
